# Editorial: The role of posttranslational modifications in polyglutamine diseases

**DOI:** 10.3389/fnmol.2023.1271226

**Published:** 2023-08-15

**Authors:** Jonasz Jeremiasz Weber, Maria do Carmo Costa, K. Matthew Scaglione, Sokol V. Todi, Huu Phuc Nguyen

**Affiliations:** ^1^Department of Human Genetics, Ruhr University Bochum, Bochum, Germany; ^2^Institute of Medical Genetics and Applied Genomics, University of Tübingen, Tübingen, Germany; ^3^Department of Neurology, Michigan Medicine, University of Michigan, Ann Arbor, MI, United States; ^4^Department of Molecular Genetics and Microbiology, Duke University, Durham, NC, United States; ^5^Department of Pharmacology, Wayne State University, Detroit, MI, United States

**Keywords:** Huntington's disease, spinobulbar muscular atrophy, spinocerebellar ataxia, phosphorylation, protein aggregation, protein clearance, protein fragmentation, ubiquitination

Polyglutamine (polyQ) diseases represent a class of rare inherited neurodegenerative disorders, originated by the expansion of glutamine-coding CAG/A trinucleotide repeats within exons of causative genes. This class of neurological conditions includes dentatorubral-pallidoluysian atrophy (DRPLA), Huntington's disease (HD), spinobulbar muscular atrophy (SBMA), and six different types of spinocerebellar ataxias (SCAs). While all mutant proteins share expanded polyQ tracts and a common propensity to form intraneuronal aggregates, their structures and cellular functions vary. This variation may explain the diversity of brain regions affected by the different disorders (Stoyas and La Spada, 2018; Lieberman et al., 2019; Bunting et al., 2022). To unravel the pathogenesis of these devastating diseases, it is crucial to have a comprehensive understanding of the molecular consequences triggered by polyQ expansion on the functional integrity of the disease proteins. Moreover, identification of disease-modifying factors is fundamental for developing strategies to treat these incurable disorders.

An important factor within the pathomechanistic complexity of polyQ disorders are posttranslational modifications (PTMs), which have manifold repercussions on the targeted disease protein. More than 400 different types of PTMs exist, including acetylation, glycosylation, lipidation, phosphorylation, non-proteasomal proteolysis, SUMOylation, and ubiquitination, which all exert profound effects on the function, localization, and stability of targeted proteins (Mann and Jensen, 2003; Ramazi and Zahiri, 2021). Consequently, by altering these properties, PTMs can also influence the toxicity and aggregation-prone nature of polyQ disease proteins, thereby significantly contributing to the progression of the disorder (Sambataro and Pennuto, 2017). In their review article, Johnson et al. summarize the most current knowledge on PTMs in all nine polyQ disease proteins. Additionally, they present information on their described proteinaceous interactors, shedding light on the significant modulatory role of both aspects in the molecular pathogenesis.

Focusing specifically on androgen receptor (AR), the disease protein of SBMA (Hashizume et al., 2020), Gogia et al. review research work analyzing the contribution of acetylation, methylation, SUMOylation, ubiquitination, and, in particular, phosphorylation of AR on the functional regulation of the protein. They also explore both the pathogenic and protective roles of these PTMs in SBMA pathogenesis. In addition, Sengupta et al. discuss in detail the importance of AR ubiquitination, including the associated enzymes and pathways behind this PTM, namely E3 ubiquitin ligases, deubiquitinases (DUBs), and the degradative ubiquitin proteasome system.

One of the most significant PTMs is proteolytic fragmentation, which irreversibly removes portions of the substrate proteins, thereby permanently altering their structure and function. Several classes of enzymes, such as caspases, cathepsin, or matrix-metalloproteinases, have been shown to cleave polyQ proteins, generating detrimental protein fragments. This process is linked to the toxic fragment hypothesis of neurodegeneration (Weber et al., 2014; Matos et al., 2017). An in-depth examination of the involvement of calpains, a class of calcium-activated proteases, was provided in a review article by Incebacak Eltemur et al. The article underscores the importance of calpains in the cleavage process as a unifying molecular pathomechanism in polyQ diseases and their targetability for the development of new therapies.

The large huntingtin protein associated with HD offers numerous sites for posttranslational modifications, and significant scientific research has focused on their physiological and HD-related pathological importance (Ehrnhoefer et al., 2011; Saudou and Humbert, 2016). Notably, the first 17 amino acids of huntingtin undergo extensive posttranslational modifications, including phosphorylation and ubiquitination, which have been linked to the regulation of mutant huntingtin toxicity and aggregation (Gu et al., 2009; Hakim-Eshed et al., 2020). In this context, Zhao et al. analyzed the consequences of removing huntingtin exon 1, which encodes the very N-terminus as well as the polyQ and polyproline stretches of the protein, in a mouse model, to assess whether its absence would adversely affect physiological characteristics. Interestingly, various mechanisms important for cellular homeostasis remained unaltered, suggesting that removal of the N-terminus of mutant huntingtin could potentially serve as a therapeutic strategy without compromising the protein's normal function.

Among all polyQ disease proteins, the Machado-Joseph disease (MJD, also known as SCA3) protein ataxin-3 holds a unique position. It not only serves as a target for PTMs but also functions as a DUB, involved in trimming polyubiquitin chains from other substrate proteins (Matos et al., 2011). As a result, posttranslational modifications of ataxin-3 and its polyQ expansion-related dysfunction have been associated with the molecular pathogenesis of MJD. In their cell-based study, Pereira Sena et al. investigated the significance of lysine residues within the ataxin-3 protein, with absence of all or presence of certain residues showing implications on the polyQ protein stability, polyubiquitin chain-binding, aggregation propensity and toxicity. The capability of ataxin-3 to bind and trim polyubiquitin chains is known to be altered by the polyQ expansion. Luo et al. analyzed changes of global ubiquitination levels in both ataxin-3 knockout and MJD cell or mouse models, showing that the absence of the wild-type protein and the presence of polyQ-expanded ataxin-3 differently impact K48- and K63-linked polyubiquitin levels. The active involvement of ataxin-3 in modulating ubiquitination and being itself a substrate for this PTM highlights the complexity of these modifications and their role in disease progression.

In conclusion, this Research Topic on posttranslational modifications in polyQ diseases underscores the crucial role that PTMs play in the molecular pathogenesis of these disorders. A thorough comprehension of how PTMs affect the physiological function and stability of disease proteins holds the potential to pave the way for the development of interventions that can restore normal cellular processes disrupted by polyQ expansions. The ability to target specific PTMs has been demonstrated in multiple preclinical studies focusing on various polyQ disorders, showing compelling results in modulating aggregation, toxicity, or clearance of mutant proteins. This offers an auspicious avenue for the discovery of novel therapeutic strategies. Thus, further advancements in this field present a great promise for improving the lives of individuals affected by these debilitating conditions.

## Author contributions

JW: Writing—original draft, Writing—review and editing. MC: Writing—original draft, Writing—review and editing. KS: Writing—original draft, Writing—review and editing. ST: Writing—original draft, Writing—review and editing. HN: Writing—original draft, Writing—review and editing.
